# Lightweight Fruit-Detection Algorithm for Edge Computing Applications

**DOI:** 10.3389/fpls.2021.740936

**Published:** 2021-10-13

**Authors:** Wenli Zhang, Yuxin Liu, Kaizhen Chen, Huibin Li, Yulin Duan, Wenbin Wu, Yun Shi, Wei Guo

**Affiliations:** ^1^Department of Information, Beijing University of Technology, Beijing, China; ^2^Institute of Agricultural Resources and Regional Planning, Chinese Academy of Agricultural Sciences, Beijing, China; ^3^Key Laboratory of Agricultural Remote Sensing, Ministry of Agriculture, Beijing, China; ^4^International Field Phenomics Research Laboratory, Institute for Sustainable Agro-Ecosystem Services, The University of Tokyo, Tokyo, Japan

**Keywords:** modern horticulture, deep learning, fruit detection, lightweight, edge devices

## Abstract

In recent years, deep-learning-based fruit-detection technology has exhibited excellent performance in modern horticulture research. However, deploying deep learning algorithms in real-time field applications is still challenging, owing to the relatively low image processing capability of edge devices. Such limitations are becoming a new bottleneck and hindering the utilization of AI algorithms in modern horticulture. In this paper, we propose a lightweight fruit-detection algorithm, specifically designed for edge devices. The algorithm is based on Light-CSPNet as the backbone network, an improved feature-extraction module, a down-sampling method, and a feature-fusion module, and it ensures real-time detection on edge devices while maintaining the fruit-detection accuracy. The proposed algorithm was tested on three edge devices: NVIDIA Jetson Xavier NX, NVIDIA Jetson TX2, and NVIDIA Jetson NANO. The experimental results show that the average detection precision of the proposed algorithm for orange, tomato, and apple datasets are 0.93, 0.847, and 0.850, respectively. Deploying the algorithm, the detection speed of NVIDIA Jetson Xavier NX reaches 21.3, 24.8, and 22.2 FPS, while that of NVIDIA Jetson TX2 reaches 13.9, 14.1, and 14.5 FPS and that of NVIDIA Jetson NANO reaches 6.3, 5.0, and 8.5 FPS for the three datasets. Additionally, the proposed algorithm provides a component add/remove function to flexibly adjust the model structure, considering the trade-off between the detection accuracy and speed in practical usage.

## Introduction

With the rapid development of computer vision techniques applied in modern horticulture applications in the recent years, fruit detection has been widely used for fruit-quality detection, ripeness identification, yield prediction, and automatic picking applications as the first step of processing. However, the research findings are not adequately extended to practical applications. For example, most of the existing high-accuracy fruit-detection algorithms cannot be deployed to field robots because the computing device in the robot has a low arithmetic power and fruit detection determines the subsequent operations to be performed by the robot (Barth, 2018; Kang et al., 2020). Therefore, there is an urgent requirement for a lightweight and a highly accurate fruit-detection algorithm for deployment in robots, to accomplish the real-time detection of fruits.

In the last few decades, many researchers have investigated fruit-detection algorithms. In most previous studies on the image processing techniques for fruit detection, manually selected features were used to encode the image data acquired using different sensors and accordingly, the fruit position was estimated. Most traditional methods for fruit image recognition involve a combination of features, such as the color, texture, and shape of the target (Burnett and Blaschke, 2003; Bulanon et al., 2009; Zhao et al., 2016). Gongal et al. (2015) reviewed traditional image processing techniques applied to the agricultural field. Blasco et al. (2003) used an algorithm based on the inter-pixel spectral response as a feature for detecting orange fruits. These traditional methods are based on the study of specific scenarios and generally have a low accuracy in real applications, where the working environment is complex. Recently, the introduction of deep learning has led to significant advances in object recognition. There are many scholars who have applied deep learning methods in agriculture, including yield estimation by detecting fruits and improving crop quality by pest and disease detection (Gao et al., 2020; Mu et al., 2020; Dhaka et al., 2021; Kundu et al., 2021). Mu et al. (2020) developed an R-CNN algorithm using Resnet-101 as the backbone network for the detection, counting, and size estimation of green tomatoes. The limitations of fruit detection in the shading and growth stages were improved, and the average detection time per image was 0.37 s at 2.7 FPS on a GTX 1060 graphics card. Koirala et al. (2019) proposed a YOLOv3-based mango-detection algorithm, MangoYOLO, that was applied to the front and rear dual images of each fruit tree, and the detection time reached 70 ms at 14.3 FPS per image in a cluster of high-performance computers. Liu et al. (2020) proposed an improved tomato-detection model, YOLO-Tomato, based on YOLOv3. The traditional rectangular bounding box was replaced with a circular bounding box to match the tomato target more accurately. The time taken to detect each image could reach 54 ms at 18.5 FPS on a GTX 1070Ti graphics card. Wan and Goudos (2020) proposed an improved Faster R-CNN network for multi-category fruit detection. The detection speed could reach 58 ms at 17.2 FPS per image on an Nvidia GTX 1060 graphics card. Fu et al. (2020) established an apple-detection algorithm using two fast neural network structures, ZFNet and VGG16, to detect apples from the original RGB and foreground RGB images and improved the detection accuracy with an average detection time of 0.181 s at 5.5 FPS per image in an Nvidia TITAN XP graphics card. Yu et al. (2019) trained a strawberry dataset using a mask R-CNN algorithm to segment strawberry fruits and assist in the visual localization of picking points for picking robots, with an average time of 0.125 s at 8.0 FPS per image, processed on a GTX 1080 graphics card. Song et al. (2019) developed and trained a Faster R-CNN model implemented using VGG16, for the all-day picking of kiwifruit. The algorithm was configured in a desktop computer with an average detection time of 0.347 s at 2.9 FPS per image detected on an Nvidia TITAN XP graphics card. Gao et al. (2020) proposed a multi-class apple detection algorithm based on Faster R-CNN. The apples were detected separately under different occlusion conditions to assist the robot in developing a picking strategy, and the detection speed of processing an image on an Nvidia TITAN XP graphics card was 0.241 s at 4.1 FPS. Although these algorithms can further improve the detection accuracy by avoiding the influence of the external environment on fruit detection, the powerful feature extraction and generalization capabilities of deep neural networks also require a large number of parameters and computational cost. However, to achieve the aim of real-time detection, the detection speed should be at least 20 FPS. Otherwise, the speed is further degraded or even unworkable when the algorithm is incorporated in the edge devices of field robots. With the increasing demand for detection performance and generalization capability, achieving real-time detection with the highest possible detection accuracy has become an important research topic. One of the most important research directions is the lightweighting of the network.

A number of studies have introduced model lightweighting in various fields, excluding agriculture. Howard et al. (2017) proposed MobileNet, a mobile model, that is based on depthwise separable convolution, instead of the traditional convolutional operations. The computational complexity is significantly reduced. An improved version of MobileNetV2 was thereafter proposed by Sandler et al. (2018) to further improve the performance of the network model, by adding an inverted residual structure with linear bottlenecks to the network. Zhang et al. (2018) proposed a lightweight neural network model, ShuffleNetV1, that ensures network performance while reducing the operational complexity through group convolution and channel shuffling. Ma et al. (2018) analyzed the relationship between the computer memory access loss time and running speed on this basis, and they also emphasized the effect of four factors, namely, the number of input and output channels of the convolutional layer, number of group operations, number of branches of the network model, and number of element-wise operations, on the speed of the overall model. Subsequently, the network model was further improved, and the ShuffleNetV2 network model was proposed. The algorithms of the YOLO series family (Redmon et al., 2016; Redmon and Farhadi, 2017, 2018; Bochkovskiy et al., 2020) have gained wide recognition in the one-stage target detection in the recent years. It uses a direct prediction of the object bounding box, combining the two phases of region proposal and object detection into one stage, and its backbone network, Darknet, can also be replaced with the other backbone networks, thereby achieving efficiency, flexibility, and an appropriate generalization performance. Among them, the performance of the Yolo-tiny series of network models has attained new highs in lightweighting. The average accuracy of yolov4-tiny on the COCO dataset exceeds that of the mainstream lightweight networks (Jiang et al., 2020). However, because the fruit size in the actual field generally varies, few being extremely small, the detection accuracy is relatively low, owing to the fact that the network structure of the lightweight model is relatively simple, with a small number of layers; therefore, the effective features that can be extracted from the target are also relatively few that causes the performance of small-sized fruit detection to be unsatisfactory. This is also one of the reasons that hinders the application of lightweight networks in the field of fruit detection.

In this study, we propose a lightweight network model based on edge devices and deploy the model on portable and powerful edge devices that can achieve a high accuracy and real-time fruit detection, and the specific issues in the existing literature that are addressed by this study are given below.

(1) To address the large number of parameters and computation cost of the existing fruit-detection networks owing to the complex structures, in this paper, a computationally efficient lightweight CSP target detection network, Light-CSPNet, is proposed for fruit detection. The accuracy and speed of fruit detection are significantly improved.
(2) To address the low detection accuracy of the existing lightweight networks owing to fewer layers and insufficient feature representation capability, this paper proposes a down-sampling method based on the variation in the feature-map size for lightweight networks. It replaces the single down-sampling strategy used in the mainstream methods. This can facilitate exhaustively utilizing the feature-map characteristics with different scales. The detection accuracy of the lightweight model is further improved.
(3) To address the problem of apparent differences in the fruit size in actual fields, this paper proposes a deep–shallow-layer fusion model that fuses features at three different scales and performs feature fusion through the multiscale fusion of the dual-attention mechanism, to strengthen the feature expression capability and substantially improve the detection accuracy for different fruit sizes.

Through experimentation, it has been shown that the network model proposed in this paper can substantially improve the detection accuracy and achieve real-time detection using edge devices. The remainder of this paper is organized as follows. Section 2 describes the proposed lightweight fruit-detection network. Section 3 discusses the related experiments performed and the corresponding results. Section 4 presents the conclusions of this study.

## Materials and Methods

In this paper, we propose a lightweight fruit-detection algorithm for edge device applications. We introduce the overall structure of the algorithm in Section 2.1, the backbone network of the network model in Section 2.2, the feature-fusion module of the network model in Section 2.3, and the detection branch of the output in Section 2.4.

### Overall Algorithm Structure

**FIGURE 1:**
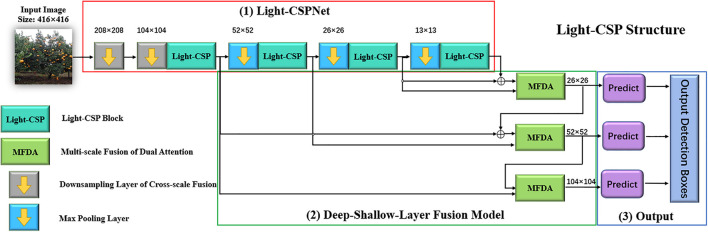
General architecture of the algorithm.

This section mainly describes the flow of our proposed algorithm, and Figure 1 shows the overall structure of the algorithm. The algorithm consists of three main steps: first, the orchard image is input to the backbone network, Light-CSPNet, to extract features; thereafter, the extracted features are passed through the deep and shallow feature-fusion module, and multiscale feature fusion is performed using the dual attention algorithm based on multiscale fusion, and finally, three shallow detection branches are output to obtain the fruit-detection results. The details of the algorithm architecture are as follows:

(1) Light-CSPNet: The backbone network is Light-CSPNet, a lightweight network for edge devices, as shown in Figure 1(1) in the red box. Cross-stage fusion enables the network model to retain the more effective gradient information expression features. There are two main features: the design of the Light-CSP block and the down-sampling method based on the variation of the feature-map size.
(2) Deep–shallow-layer fusion model: Three shallow branches with different scales were designed to extract small targets of the fruit class based on prior knowledge. In the feature-fusion part, the feature information of three different depths and sizes in the backbone network is first extracted. Thereafter, feature fusion is performed through a dual attention mechanism based on the multiscale fusion function to further enhance the feature representation and improve the detection accuracy, as shown in the green box in Figure 1(2).
(3) Shallow detection branches: The three different scales of feature maps obtained in the previous section were used to construct three different detection branches for the target fruit detection. The non-maximum suppression algorithm (NMS) (Girshick et al., 2014) was used to remove the duplicated frames and those with low confidence scores. The obtained results are the final fruit-detection results, as shown in the blue box in Figure 1(3).

### Backbone Network

The backbone network, Light-CSPNet, is based on CSPNet (Wang C. Y. et al., 2020), with the features detailed below:

(1) To address the problem of the high computational cost of real-time fruit detection, the internal structure of the blocks used in the original CSPNet at different scales is lightened and replaced with Light- blocks for extracting features, thereby avoiding time-consuming detection.
(2) To address the problem in the small-sized fruit detection, a multi-strategy down-sampling method based on the variation of the feature-map size is used. Different down-sampling methods are used according to the size of the down-sampling rate to improve the feature extraction ability.

**FIGURE 2:**
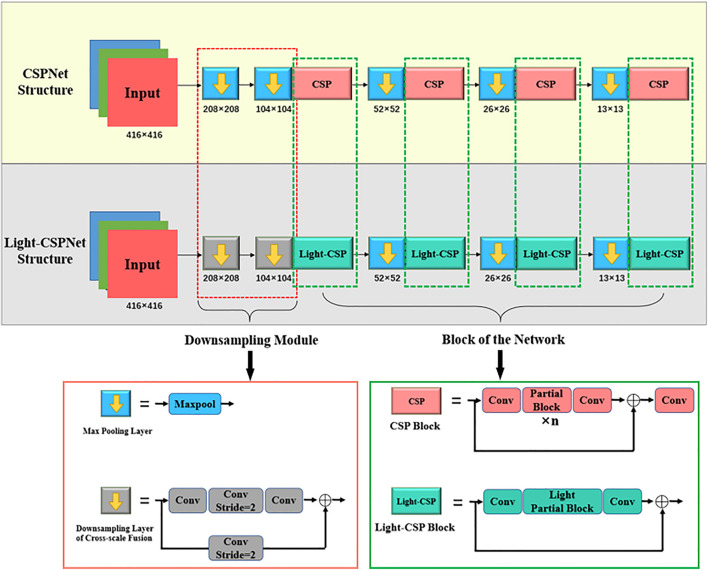
Comparison of CSPNet and Light-CSPNet backbone networks.

Figure 2 shows the comparison of the backbone network structure between the conventional CSPNet and the proposed Light-CSPNet. The red and green dashed boxes are the parts of the changes made in this study to the internal structural parts of the down-sampling method and the convolutional block, respectively. In this section, we present the proposed improved parts, where the Light-CSP block part in the green box is introduced in Section 2.2.1, and the down-sampling part based on the change in the feature-map size in the red box is introduced in Section 2.2.2.

#### Light-CSP Block

In the conventional convolutional neural network architectures, such as ResNet (He et al., 2016) and DenseNet (Huang et al., 2017), the output of each layer is composed of the convolutional operation of the layer itself and the output of all the previous layers. However, this results in a gradient flow in the k^th^ layer of the model while updating and using the weights by retracing from the 1st layer to the k^th^ layer each time, leading to repeated learning of redundant information. In recent years, researchers have also investigated improving the learning capability and parameter information utilization of the model (Ma et al., 2018; Zhu et al., 2018; Wang C. Y. et al., 2020), where CSPNet (Wang C. Y. et al., 2020) uses cross-stage partial concatenation to map the output x_k–1_ of the (k-1)^th^ layer convolution operation into two parts in the next layer. It merges them after passing them through a cross-stage hierarchy, such that, one of the parts is updated with the gradient, while the other part keeps the original gradient information unchanged. This ensures that the propagated gradient information maintains a large correlation difference, thereby reducing the gradient reuse problem. CSPNet also achieved state-of-the-art test results on the MS COCO target detection dataset. The network structure of CSPNet can be expressed as follows:


(1) $$x_{k}=M\{\left[x_{k-1}^{\prime },T\left(F\left(x_{k-1}^{\prime \prime }\right)\right)\right]$$

where *x*_*k–1*_ is the input of this layer that is divided into two channels after passing it through a convolution layer and can be expressed as $x_{k-1}=\left[x_{k-1}^{\prime },x_{k-1}^{\prime \prime }\right]$.*T* is the transition function used to truncate the gradient flow in *x*_*k*_(*k* = 1,*k* = 2).*M* denotes a 1 × 1 convolution operation for integrating the two separated parts while controlling the number of channels.

However, owing to the large parameters and a high computational cost, CSPNet exhibits a long detection time. Therefore, it is not suitable for deployment in field robots.

**FIGURE 3:**
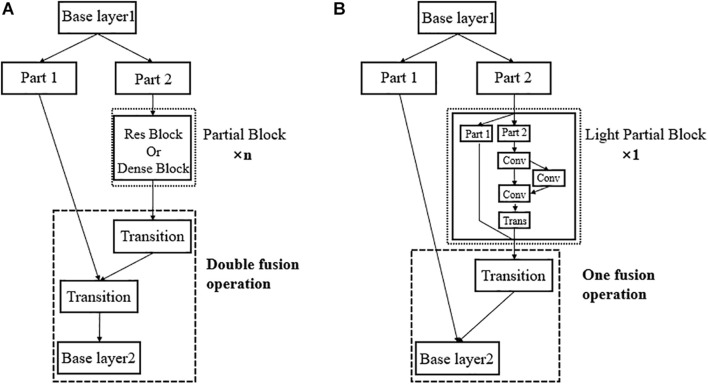
Using the excellent structural design of CSPNet, we propose a lightweight network model, Light-CSPNet. Here, the n partial block in the original network is replaced with a lightweight, specifically designed light partial block, and the double fusion operation is replaced with one fusion operation to further improve the network operation speed. **(A)** Shows the network architecture of CSPNet; **(B)** Shows the network architecture of the proposed Light-CSPNet.

In this paper, we propose Light-CSPNet to shorten the detection time, while ensuring the detection accuracy. As shown in Figures 3A,B, we replace the large number of Res blocks or Dense blocks stacked in the partial block of the CSPNet with a miniature cross-stage network structure consisting of only three convolutional layers, the light partial block. And we reduce a partial transition layer to reduce (with fusion last strategy) the computational effort.

The n convolutional modules stacked in the partial block part of the CSPNet with a nested miniature cross-stage network structure consisting of only a few convolutional layers has been replaced and is represented as


(2) $$x_{k}=M\,\left\{\left[x_{k-1}^{\prime },T\,\left[F\,\left(y_{k-1}^{\prime },T^{\prime }\,\left(F\,\left(y_{k-1}^{\prime \prime }\right)\right)\right)\right]\right]\right\}$$

where $y_{k-1}^{\prime }$ and $y_{k-1}^{\prime \prime }$ are the two different channels from $x_{k-1}^{\prime \prime }$ that is divided before entering the next convolutional layer. They can be expressed as $x_{k-1}^{\prime \prime }=\left[y_{k-1}^{\prime },y_{k-1}^{\prime \prime }\right],$ and *T*′ is the new transition function that truncates the gradient flow in the previous convolutional layer.

By switching and reintegrating the channel streams, we reduce the large amount of duplicated gradient information that allows the gradients to maintain appropriate correlation differences during propagation and further reduces the amount of computation and number of parameters per block in the network. Using this method, we eliminate the requirement of a high computational cost in large networks, such as CSPNet, and improve the speed and accuracy of fruit detection in real-time applications.

**FIGURE 4:**
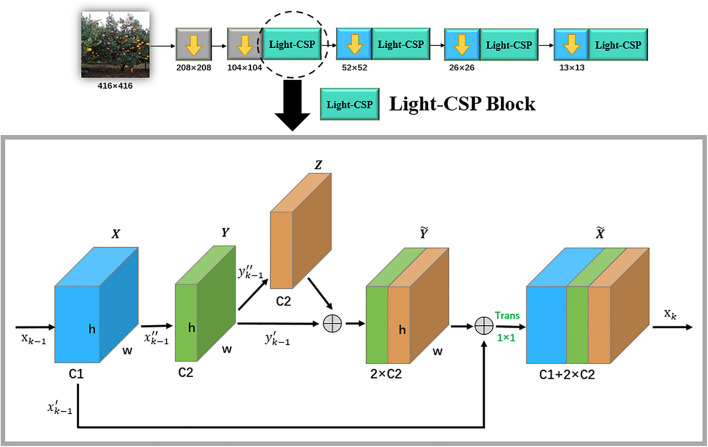
Internal flow diagram of each Light-CSP block in the backbone network.

Figure 4 shows the specific internal flow chart of each block with the corresponding scale in Light-CSPNet that can be divided into the following four steps:

(1) The output, *x*_*k–1*_, of the previous layer is used as the input of this layer. The output *X* after passing a 3 × 3 × C1 convolutional layer is mapped into two parts $x_{k-1}^{\prime },x_{k-1}^{\prime \prime }$ for separate operational processing.
(2) $x_{k-1}^{\prime }$ is kept constant. After passing $x_{k-1}^{\prime \prime }$ through another 3 × 3 × C2 convolutional layer to output *Y*, it is again mapped again into two parts of $y_{k-1}^{\prime }\,a\,n\,d\,y_{k-1}^{\prime \prime }$.
(3) After passing $y_{k-1}^{\prime \prime }$ through a 3 × 3 × C2 convolutional layer with output*Z*, it is thereafter fused with$y_{k-1}^{\prime }$ into the $\tilde{Y}$ of dimension 2 × C2.
(4) $\tilde{Y}$ is fused with the previous $x_{k-1}^{\prime }$, and thereafter, the 1 × 1 convolution operation is performed to double the dimension to$\tilde{X}$, whose dimension is C1 + 2 × C2. Finally, the result is input as *x_k_* in the next section.

In this framework, we use a nested two-span phase network structure to propagate the gradient information. Compared with stacking n Res blocks and Dense blocks, the computational and parametric quantities of this network are significantly reduced, further improving the operational efficiency.

#### Down-Sampling Method Based on the Variation of Feature-Map Size

In this paper, we propose a down-sampling method based on the variation of the feature-map size to address the problem encountered while employing the conventional single down-sampling method that cannot effectively retain the various scales of feature maps at different down-sampling rates, and accordingly, our proposed method improves the detection accuracy.

In the field of fruit detection, the size of many fruits as observed in the images are small. After several iterations of down-sampling, the image size of a target fruit is only of a few pixels. In such scenarios, if the conventional convolution with stride is 2 and the activation function, ReLU, is used for down-sampling, the relationship information between the neighboring pixels will be significantly lost, resulting in a degradation of the detection performance that is not ideal for small-sized target detection.

Luo et al. (2016) studied the relationship between feature maps and receptive field size at different scales and proposed that the feature maps at different scales contain different feature information. If a single down-sampling method is used, then a large amount of useful information will be ignored and the detection accuracy will be reduced. Based on this, we adopt a down-sampling method in backbone network based on the size variation of feature map, which switches the down-sampling methods according to the different down-sampling rate.

(1) When the down-sampling rate is low, the image size is large, the receptive field is small, and the extracted features are all low-level texture and color features. A down-sampling method based on the cross-scale fusion function is proposed to remedy the problem encountered, owing to the inability to simultaneously retain the effective information of complex images and the information between neighboring pixels in the conventional down-sampling methods.
(2) When the down-sampling rate is high, the image size is small, the receptive field is large, and the extracted features are all high-level semantic features. At this time, using the max-pooling method, the relationship between adjacent pixels can be preserved, while still maintaining the translation invariance of the image. This algorithm can improve the detection accuracy of small-sized target fruits, without increasing the computational effort.

**FIGURE 5:**
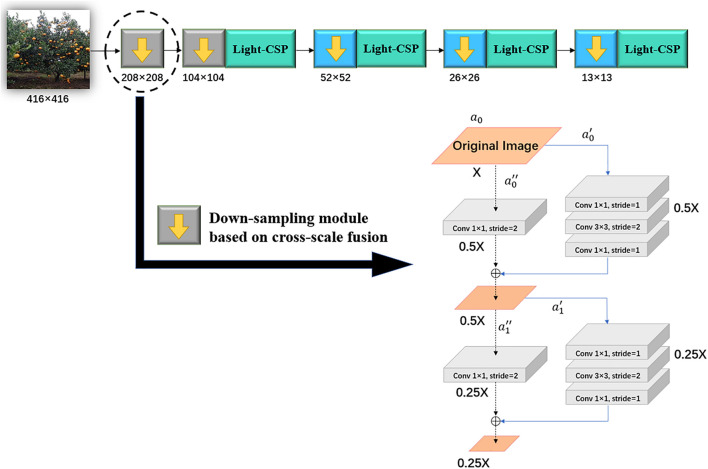
Network structure of down-sampling method based on cross-scale fusion function.

The specific structure of the down-sampling method based on the cross-scale fusion function used at low down-sampling rates is shown in Figure 5. Because this down-sampling method is used to extract features in shallow networks and shallow features are prone to gradient disappearance, the general idea of the algorithm is based on the design concept of residual blocks in ResNet. (1) First, the original image *a_0_* with Scale × 1 is input and mapped into two channels that can be expressed as$a_{0}=\left[a_{0}^{\prime },a_{0}^{\prime \prime }\right]$. (2) $a_{0}^{\prime }$ is down-sampled through a convolution operation with a stride of 2 in a convolution layer with a convolution kernel of 3 × 3, to ensure that the computational effort is not significantly increased while using convolution for feature extraction. The scale of the feature-map output from this channel becomes half of that of the original image: Scale × 0.5. (3)$a_{0}^{\prime \prime }$ is convolved by a convolution kernel with a 1 × 1 kernel and a stride of 2. At this point, the scale of the feature-map output from this channel also becomes half of that of the original one: Scale × 0.5. (4) $a_{0}^{\prime }$ and $a_{0}^{\prime \prime }$ channels are concatenated through a down-sampling layer of cross-scale fusion to complete a down-sampling operation. The image size is reduced from Scale × 1 to Scale × 0.5. Two such down-sampling methods based on the cross-scale fusion function are performed in the shallow network of the input image to ensure that the training process reduces the information loss between neighboring pixels while achieving effective extraction of the shallow features.

By employing this down-sampling strategy based on the variation of the feature-map size, we can flexibly change the down-sampling method according to the down-sampling rate. The use of a down-sampling method based on a cross-scale fusion function at low down-sampling rates can preserve useful information in complex images and improve the efficiency of gradient utilization in shallow networks. The max-pooling operation for down-sampling at high down-sampling rates can reduce the loss of information between neighboring pixels and completely extract the deep semantic information in the features.

#### Internal Parameters of Light-CSPNet Network Architecture

The internal parameters of the network architecture of Light-CSPNet are shown in Table 1. It contains two down-sampling methods based on a cross-scale fusion function and three maximum pooling modules with a down-sampling method based on the feature-map size variation and three convolution modules in the Light-CSP Block for lightweight feature extraction. Each layer was followed by batch normalization and a Leaky ReLU activation function.

**TABLE 1:** Internal parameters of the network architecture of Light-CSPNet.

Block name	Filter shape	Input	Output
Down-sampling method based on cross-scale fusion	1 × 1 × 32 3 × 3 × 32 (Stride = 2) 1 × 1 × 32	416 × 416 × 3	208 × 208 × 32
Down-sampling method based on cross-scale fusion	1 × 1 × 64 3 × 3 × 64 (Stride = 2) 1 × 1 × 64	208 × 208 × 32	104 × 104 × 64
Light-CSP block	3 × 3 × 64 3 × 3 × 32 3 × 3 × 32	104 × 104 × 64	104 × 104 × 64
(Transition filter)	1 × 1 × 64	104 × 104 × 64	104 × 104 × 128
Maxpool	Stride = 2	104 × 104 × 128	52 × 52 × 128
Light-CSP block	3 × 3 × 128 3 × 3 × 64 3 × 3 × 64	52 × 52 × 128	52 × 52 × 128
(Transition filter)	1 × 1 × 128	52 × 52 × 128	52 × 52 × 256
Maxpool	Stride = 2	52 × 52 × 256	26 × 26 × 256
Light-CSP block	3 × 3 × 256 3 × 3 × 128 3 × 3 × 128	26 × 26 × 256	26 × 26 × 256
(Transition filter)	1 × 1 × 256	26 × 26 × 256	26 × 26 × 512
Maxpool	Stride = 2	26 × 26 × 512	13 × 13 × 512
Light-CSP block	3 × 3 × 512 3 × 3 × 256 3 × 3 × 256	13 × 13 × 512	13 × 13 × 512
(Transition filter)	1 × 1 × 512	13 × 13 × 512	13 × 13 × 18

### Feature-Fusion Module

**FIGURE 6:**
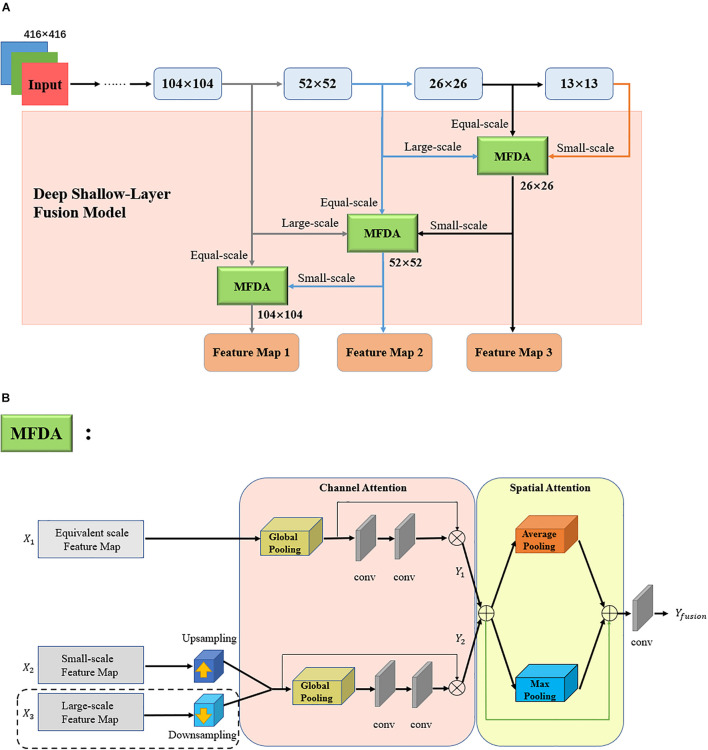
**(A)** Overall architecture of the deep–shallow-layer fusion model. **(B)** Flow of multiscale fusion of dual attention (MFDA) algorithm.

In this paper, a three-channel deep–shallow layer fusion model is proposed. The feature maps of different scales are fed into a multiscale fusion of dual attention (MFDA) algorithm for feature fusion, to further improve the detection accuracy. The target information focused on deep and shallow networks will be different in solving machine-vision tasks with convolutional neural networks. Feature fusion of different feature maps can effectively improve the detection accuracy and generalization ability of the model in object detection (Liu et al., 2016; Tsung-Yi et al., 2017; Kirillov et al., 2019; Wang W. et al., 2020). In the field of horticulture, for example, in an orchard, the size of the fruits varies. Therefore, a significant difference in the target size and an inconsistent fruit resolution will be observed. If only shallow features are used to enhance the small-sized target detection capability, it will lead to the loss of the high-level semantic features. This can easily cause gradient disappearance, and the improved utilization of the feature-map information of different layers for fruit detection at different scales is the key to this task. Based on this, the deep–shallow-layer fusion module in this study extracts feature information from three channels: small-scale, equal-scale, and large-scale channels. Thereafter, the three feature maps are input to the MFDA module for feature fusion, to improve the feature representation at different scales. The overall architecture of the deep–shallow-layer fusion model and the flow of the MFDA algorithm are shown in Figures 6A,B, respectively.

The pink area in Figure 6A is the proposed deep–shallow-layer multiscale fusion module. The backbone network, Light-CSPNet, convolves the input raw image to obtain features with different scales as the down-sampling rate increases. This module up-samples the deep semantic features and down-samples the shallow texture features. Thereafter, it is concatenated with the feature maps at equal scales into the MFDA algorithm to highlight the feature expressions and improve the fruit feature expressions at different scales as well as the detection of small targets. Only equal-scale and small-scale features were fused in the 104 × 104 branch. The final feature maps of the three shallow branches were used for subsequent detection.

The MFDA algorithm inputs features from three channels and enhances the correlation between features using the channel attention mechanism and the spatial attention mechanism successively. The algorithm flow is illustrated in Figure 6B. The algorithm is divided into two parts: (1) First, the equal-scale feature map *X_1_*, the small-scale feature map *X_2_*, and the large-scale feature map *X_3_*, obtained by up-sampling and down-sampling, are input into the channel attention module. Thereafter, the enhanced features, *Y_1_* and *Y_2_*, are connected by dimension to obtain *Y*. Finally, the feature map *Y* is input to the spatial attention module to obtain *Y*_*fusion*_. It is worth noting that the 104 × 104 branch does not necessitate down-sampling the large-scale feature map of *X_3_*.

Our proposed deep–shallow layer fusion model can enhance the correlation between the color and texture information of the shallow layer and the semantic information of the deep layer at different scales of the feature pyramid and comprehensively utilize the features extracted from each layer through convolutional neural networks. The correlation between the features of each layer is improved, and the detection accuracy of the targets at different scales is significantly improved.

### Detection Branch

In this study, we address the feature maps obtained from the output of the deep–shallow layer fusion model at three different scales. Thereafter, a separate prediction branch is constructed for each feature map to detect the target fruits at that specific scale. We replaced the deep 13 × 13 scale branches with shallow 104 × 104 scale branches to improve the detection accuracy of small-sized target fruits. In addition, the K-means target border clustering algorithm in YOLOv3 is used in this study to assign three anchors to each prediction branch for focusing on target detection at small target scales in the fruit class. Finally, the output feature vectors of the three prediction branches are combined. Prediction borders with lower confidence threshold scores were used. The NMS algorithm was applied to reject the overlapping borders of the same target to obtain the final fruit-detection results.

## Experiment

In this section, we qualitatively and quantitatively evaluated the proposed algorithm. To meet the requirements of multi-variety heterogeneous collection in practical applications, the algorithm for different fruit-detection datasets was tested in this study. Furthermore, various conventional network models were compared horizontally to evaluate the performance of the different network architectures for the detection of different fruit datasets. A GEFORCE GTX 1080Ti GPU, Intel i7 8th CPU computer was the hardware device used in this experiment to train a mature model for the overall algorithm training. The model is also deployed in portable, well-performing edge devices to test the detection accuracy and detection speed. The tests were conducted on three edge devices of the NVIDIA Jetson series, with different prices and algorithm powers: NVIDIA Jetson Xavier NX, NVIDIA Jetson TX2, and NVIDIA Jetson NANO.

### Dataset Introduction

#### Orange Dataset

**FIGURE 7:**
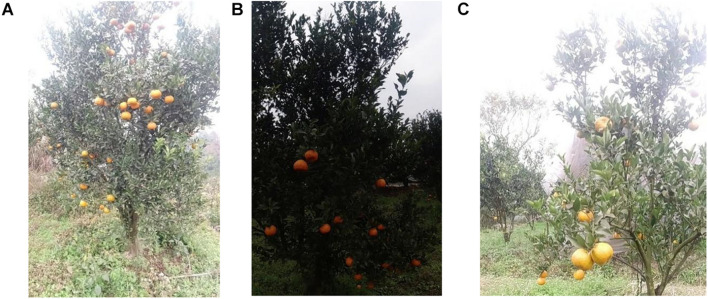
**(A)** Shows an image of small-sized citrus fruits taken in sufficient light condition. **(B)** Shows an image of small-sized citrus fruits taken in poor light condition. **(C)** Shows an image of medium-sized citrus fruits.

The orange dataset comprised of the data obtained from an orange orchard in the Sichuan Province, China, where most of the fruits were ripe and yellow. The data were collected through a standard orchard manner by controlling a robotic cart that travels through the orchard and by controlling the angle and position of the shot using a DJI Osmo Action camera mounted on the cart. The trolley must travel at a constant speed parallel to the direction of the trees, and the camera is set up at an angle of 90° to the direction of the tree growth. Simultaneously, to ensure the complexity and diversity of the dataset, data were collected from random locations in the orange orchards under a variety of leaf shading and fruit stacking conditions on different days and in different seasons. To ensure the effectiveness and practicality of fruit monitoring, the dataset contains images of orange fruits of different sizes for several different fields. Figure 7A shows the small-sized target images taken when the distance between the rover and the trees in the orchard is such that both the top of the orange tree canopy and the bottom of the tree trunk can be included in the image. Figure 7B shows an image of a small-sized target captured in an overcast scene with insufficient light intensity. Figure 7C shows an image of a medium-sized target taken at a vertical height covering only the area from the top to the bottom of the tree canopy. We have made the dataset public and uploaded it to the GitHub link^1^. The robustness and generalization ability of the algorithm can be effectively improved by training and testing the images at different scales and shooting angles.

#### Tomato Dataset

**FIGURE 8:**
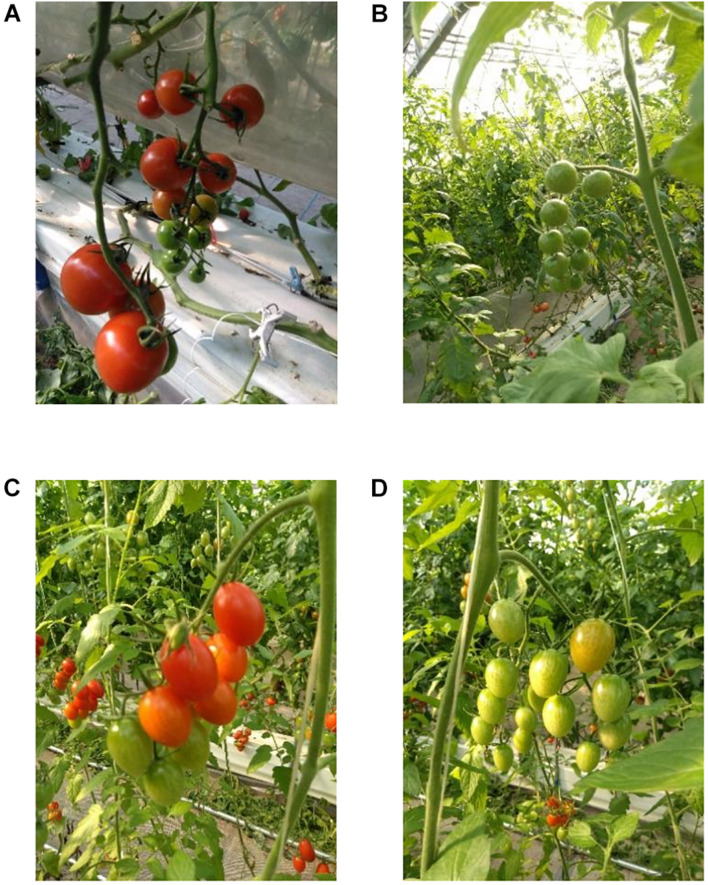
**(A)** Shows an image of large ripe tomato. **(B)** Shows an image of large immature tomato, **(C)** shows an image of small ripe tomato, and **(D)** shows an image of small immature tomato.

The tomato dataset was obtained from the public dataset Laboro Tomato^2^. It contains images of tomato fruits at different stages of growth. The dataset was obtained from a farm using two cameras simultaneously and included two subsets of tomatoes, large- and small-sized tomatoes. Each subset contains images of various fruit colors and sizes acquired under complex scenarios (background and overlapping), as shown in Figure 8. An image of the large ripe tomatoes is shown in Figure 8A. The images of an immature large tomato, a ripe small tomato, and an immature small tomato are shown in Figures 8B–D, respectively.

#### Apple Dataset

**FIGURE 9:**
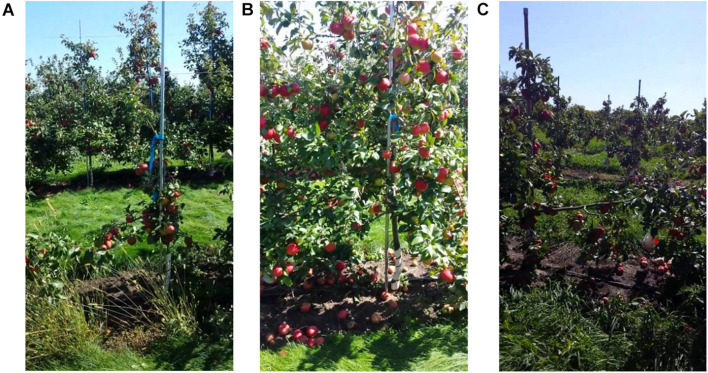
**(A)** Shows an image of ultra-small-sized apples. **(B)** Shows an image of red apples under sufficient light condition. **(C)** Shows an image of red fruit against a backlit scene.

The apple dataset is obtained from a public apple dataset, MinneApple dataset (Häni et al., 2019). The image collection contained 17 different rows of apple trees including different species of apple trees. Images were acquired using a Samsung Galaxy S4 cell phone. During data collection, video footage was acquired by facing the camera horizontally on one side of a row of trees and moving along the trees on foot at a speed of approximately 1 m/s; moving the camera at a low speed mitigates motion blur effects. One image was acquired every five frames. Data, including apple targets under various light conditions, such as, from the sunny or shady side of the tree rows, were collected over a 2-year time span on different days, as shown in Figure 9. The MinneApple dataset is based on the images of apples acquired from arbitrary angles and scenes in a highly chaotic environment that is highly demanding, in terms of the algorithm performance. The acquisition method of this dataset is similar to the method of agricultural robots capturing fruit images that is highly suitable for the application of the algorithm proposed in this study.

Figure 9A shows images of ultra-small-sized apple targets captured in a remote scene. Figure 9B shows an image of a red apple under smooth light. Figure 9C shows an image of a red fruit against a backlit scene.

### Training Strategies

We trained the different datasets separately. Finally, several different detection models were obtained. Moreover, the images were randomly split into training and test sets at a ratio of 7:3. Few of the key training parameters included the following: the gradient was optimized using the SGD optimizer, the momentum was set to 0.9, the weight decay was set to 0.0005, the initial learning rate was set to 0.01, the batch size was set to 8, and the epoch was set to 30,000. The training is done by randomly selecting a scale at certain iterations. The network was trained by compressing the original images by several scales of 320, 416, and 512 in equal proportions. Images of different scales were used as the input and selected randomly at regular intervals for training. The trained model is robust and can accept any size of image as input. The problem of low accuracy of small-sized target detection can be effectively improved (He et al., 2015). For data augmentation, Mosaic data augmentation (Yun et al., 2019) was chosen to increase the number of samples and improve the small-sized target detection ability.

### Evaluation Metrics

In this study, the precision (P) and recall (R) at the equilibrium point, as well as the average detection precision (AP), detection speed (FPS), number of model parameters (Params), floating point operations per second (FLOPs), and weight size (Weights) are used as the evaluation metrics to assess the accuracy of the target-detection performance, where the IoU threshold is set to 0.5 that is used to quantify the model accuracy, balance precision, and recall performance. Here, the *P*-value can be calculated using Eq. 4, and the *R*-value can be calculated using Eq. 5:


(3) $$P=\frac{T\,P}{T\,P+F\,P},$$

(4) $$R=\frac{T\,P}{G\,T},$$

where TP represents the number of fruits with correct detection and classification results and FP represents the number of samples that incorrectly detect non-fruit targets as fruits in the fruit dataset. GT represents the number of fruit samples obtained using the manual annotation methods.

Average detection precision is used as a standard measure to evaluate the sensitivity of the network in detecting target fruits. This metric reflects the overall performance of the network and the physical meaning is the area of the P-R curve that is the average of the *P*-value as the *R*-value varies in the interval 0–1 and can be calculated using Eq. 6:


(5) $$A\,P=\int_{0}^{1}P_{\left(R\right)}\,dR,$$

where *P*_*(R)*_ represents the P, and *P* is a function of the *R* at different confidence levels. Because the performance comparison results of P and R obtained at different confidence levels are different, this study mainly determines the performance of the different networks based on the value of AP. The higher the AP value, the better is the network performance. The rest of the evaluation values will be provided in the paper for further study and analysis. For the lightweight model, this study uses frames per second (FPS) to evaluate the detection speed of the algorithm, and its calculation can be expressed by Eq. 7:


(6) $$F\,P\,S=\frac{N}{t_{N}},$$

where *t_N_* represents the total time taken by the model to detect N images. The number of parameters of the network model (Params) can be used to measure the complexity of the network model. The larger the size and number of convolution kernels, the larger is the number of parameters. The number of FLOPs indicates the speed of the network model. The evaluation metric, Weights, was used to measure the size of the network model. These three metrics were used to determine whether the network model was sufficiently light.

### Comparative Analysis of Experimental Results

#### Comparison Experiments

The purpose of this experiment is to investigate the performance of the proposed lightweight network model for fruit detection. Moreover, the experiment verifies whether the algorithm can perform real-time detection on edge devices to be deployed in robots. Therefore, all test results and evaluation metrics of this experiment are tested on the edge devices. Three different edge devices are used in this experiment: NVIDIA Jetson Xavier NX, NVIDIA Jetson TX2, and NVIDIA Jetson NANO. We tested our proposed algorithm on orange, tomato, and apple datasets; thereafter, we experimentally compared the single-stage mainstream YOLO series algorithms mentioned above. The results are presented in Tables 2, 4 respectively, where the values in bold indicate the method proposed in this study.

**TABLE 2:** Test results for the orange dataset.

Model	P	R	AP	Params (M)	FLOPs (G)	Weights (M)	FPS		
	
							NX	TX2	NANO
YOLOv3	0.88	0.86	0.905	61.52	116.3	492.8	8.0	4.7	2.0
YOLOv4	0.862	0.877	0.911	99.2	165.7	794.8	4.5	3.1	1.3
YOLOv3-tiny	0.786	0.788	0.793	8.66	9.7	69.5	37.0	18.5	8.5
YOLOv4-tiny	0.853	0.792	0.817	6.06	13.2	48.7	32.2	16.9	13.9
**Light-CSPNet (Proposed)**	**0.856**	**0.901**	**0.930**	**5.96**	**27**	**48**	**21.3**	**13.9**	**6.3**

**TABLE 3:** Test results for the tomato dataset.

Model	P	R	AP	Params (M)	FLOPs (G)	Weights (M)	FPS		
	
							NX	TX2	NANO
YOLOv3	0.847	0.804	0.803	61.52	116.3	492.8	8.1	5.0	1.7
YOLOv4	0.831	0.777	0.774	99.2	165.7	794.8	4.6	5.4	2.0
YOLOv3-tiny	0.716	0.718	0.728	8.66	9.7	69.5	40.0	16.7	5.5
YOLOv4-tiny	0.639	0.709	0.702	6.06	13.2	48.7	52.6	20.4	6.8
**Light-CSPNet (Proposed)**	**0.804**	**0.804**	**0.847**	**5.96**	**27**	**48**	**24.8**	**14.1**	**5.0**

**TABLE 4:** Test results for the apple dataset.

Model	P	R	AP	Params (M)	FLOPs (G)	Weights (M)	FPS		
	
							NX	TX2	NANO
YOLOv3	0.835	0.821	0.822	61.52	116.3	492.8	8.2	5.0	2.3
YOLOv4	0.749	0.722	0.751	99.2	165.7	794.8	7.5	5.2	2.6
YOLOv3-tiny	0.686	0.679	0.662	8.66	9.7	69.5	50.0	16.1	9.9
YOLOv4-tiny	0.629	0.596	0.583	6.06	13.2	48.7	43.5	26.3	14.3
**Light-CSPNet (Proposed)**	**0.812**	**0.792**	**0.850**	**5.96**	**27**	**48**	**21.7**	**14.5**	**8.5**

**FIGURE 10:**
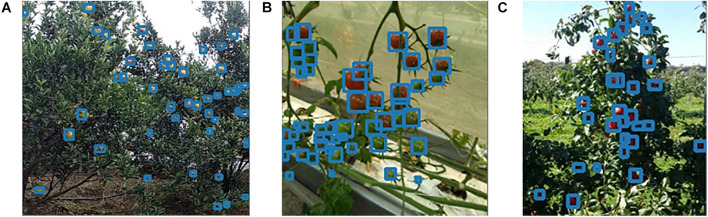
Examples of the results of detection algorithms tested on different datasets. **(A)** Citrus dataset; **(B)** tomato dataset; **(C)** apple dataset.

As shown in the table, the proposed algorithm can achieve the accuracy of 0.93, 0.847, and 0.85 for the orange, tomato, and apple datasets, respectively. All three metrics achieved the state-of-the-art (SOTA) performance. Our algorithm is sufficiently lightweight, based on the three metrics, the number of parameters, FLOPs, and weight size. Both the number of parameters and weight size were the smallest among the compared network models. Moreover, the computational FLOPs are larger than that for the YOLOv3-tiny and YOLOv4-tiny network models because the proposed network is designed with few relatively complex network structures for an improved propagation of the gradients, while retaining the ability to improve the feature representation. When deploying the proposed algorithm in Jetson Xavier NX, the detection speed reaches up to 21.3, 24.8, and 21.7 FPS for the three datasets (higher than 20) and the purpose of real-time detection is achieved. The detection speed on Jetson TX2 can reach 13.9, 14.1, and 14.5 FPS, and the detection speed on Jetson NANO can reach 6.3, 5.0, and 8.5 FPS. We belief that this experiment can provide a reference basis for agricultural practitioners intending to deploy algorithms on edge devices. It is worth noting that few frameworks and algorithms can further improve the inference speed of object detection algorithms. If GPU acceleration is applied, the detection speed will be further improved by a large factor, nonetheless the difference between the compared algorithms and the overall trend does not change. Therefore, this study does not use GPU acceleration and model quantization to further improve the inference speed in the detection speed test. Figure 10 shows the visualization of test results of our detection algorithm tested on different datasets more intuitively, where the fruit targets at different scales in the image can be detected accurately.

#### Ablation Experiment

To verify the improvement of each module proposed in this paper for detection performance, component ablation experiments were conducted to compare the performance in this study. In the initial case, Light-CSPNet is used as the backbone network, conventional maximum pooling layer for down-sampling, and feature pyramid networks (FPN) for feature fusion. In this experiment, we tested the performance improvement of the network by adding the MFDA module and replacing the traditional maximum pooling layer with a down-sampling method based on the variation of the feature-map size and replacing the FPN feature-fusion module with a deep–shallow-layer feature-fusion module, respectively. This experiment compares the respective AP and the detection speed (FPS) for each dataset, as shown in Tables 5, 7 respectively.

**TABLE 5:** Component ablation experiments on the orange dataset.

Method	MFDA	Down-sampling method (proposed)	Deep–shallow-layer fusion	AP	FPS		
	
					NX	TX2	NANO
Light-CSPNet-1				0.894	25.1	15.4	6.7
Light-CSPNet-2	√			0.917	22.2	14.9	6.5
Light-CSPNet-3	√	√		0.928	21.4	14.1	6.4
Light-CSPNet-4	√	√	√	0.930	21.3	13.9	6.3

*The three algorithms, MFDA module, down-sampling method based on the variation of the feature-map size, and deep–shallow-layer fusion module, are compared to improve the algorithm performance.*

**TABLE 6:** Component ablation experiments on the tomato dataset.

Method	MFDA	Down-sampling method (proposed)	Deep–shallow-layer fusion	AP	FPS		
	
					NX	TX2	NANO
Light-CSPNet-1				0.802	28.6	15.6	6.1
Light-CSPNet-2	√			0.815	25.6	15.2	5.8
Light-CSPNet-3	√	√		0.829	25.1	14.3	5.6
Light-CSPNet-4	√	√	√	0.847	24.8	14.1	5.0

*The three algorithms, MFDA module, down-sampling method based on the variation of the feature-map size, and deep–shallow-layer fusion module, are compared to improve the algorithm performance.*

**TABLE 7:** Component ablation experiments on the apple dataset.

Method	MFDA	Down-sampling method (proposed)	Deep–shallow-layer fusion	AP	FPS		
	
					NX	TX2	NANO
Light-CSPNet-1				0.820	27.8	15.9	11.1
Light-CSPNet-2	√			0.825	25.1	15.6	10.3
Light-CSPNet-3	√	√		0.829	21.8	14.9	9.1
Light-CSPNet-4	√	√	√	0.850	21.7	14.5	8.5

*The three algorithms, MFDA module, down-sampling method based on the variation of the feature-map size, and deep–shallow-layer fusion module, are compared to improve the algorithm performance.*

**FIGURE 11:**
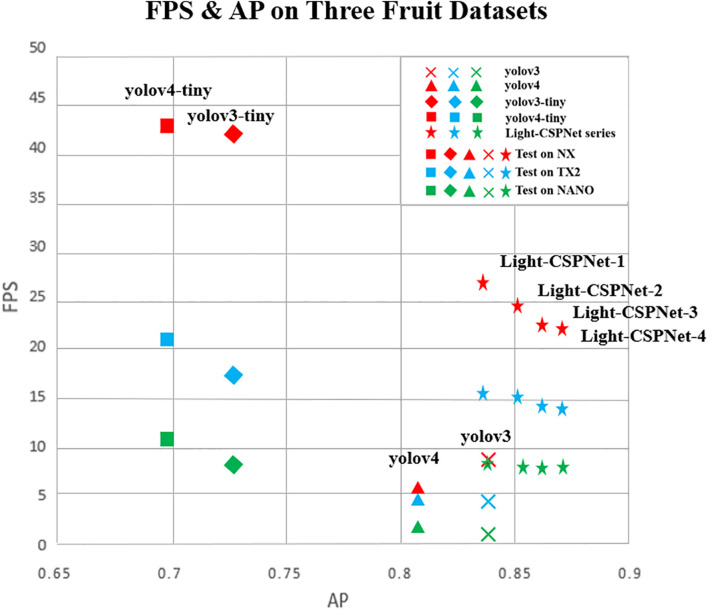
Comparison of the average AP and detection speed performance metrics of different algorithms on the three datasets of orange, tomato, and apple.

It can be concluded from the table that further improvement in the fruit-detection accuracy can be achieved by introducing the dual-attention multiscale fusion module, the down-sampling method based on the variation of the feature-map size, and the deep–shallow-layer fusion module. We tested three different edge devices. The dual-attention multiscale fusion module improves the average precision by 2.3, 1.3, and 0.5% for the orange, tomato, and apple datasets, respectively; the down-sampling method based on the variation of the feature-map size improves the average precision by 1.1, 1.4, and 0.4%, respectively, and the deep–shallow-layer fusion module improves the average precision by 0.2, 1.8, and 2.1%, respectively, with different degrees of average precision improvement for each component in different datasets. Each component brings different degrees of improvement in accuracy for different datasets. In practice, different components can be added or removed depending on the specific speed and accuracy requirements. In this study, the average detection performance of the network model with different components was compared with that of the mainstream YOLO series network model for three datasets: orange, tomato, and apple. The results are shown in Figure 11.

To distinguish the three edge devices, NX, TX2, and NANO, the different symbols in the figure represent the different algorithm results, and the different colors represent the tests performed on the different edge devices, where red indicates the tests performed on NX, blue indicates the tests performed on TX2, and green indicates the tests performed on NANO. Light-CSPNet-1, Light-CSPNet-2, Light-CSPNet-3, and Light-CSPNet-4 are the network models use only the backbone network proposed in this paper and adding the dual-attention module for multiscale fusion, the down-sampling method based on the variation of the feature-map size, and the deep–shallow-layer fusion module, respectively, can enhance the performance.

From the comparison of the different algorithms in Figure 11, we conclude that: (1) using only the proposed backbone network without adding any components can achieve an average detection accuracy close to that of the YOLOv3 network model. The detection speed is much faster than that of YOLOv3 and YOLOv4. (2) YOLOv3-tiny and YOLOv4-tiny are approximately twice as fast as the algorithm proposed in this paper because the network structure of the algorithm in this study is relatively complex for an improved detection of small fruit targets. (3) The detection accuracy of the algorithm increases with the addition of different components, nonetheless there is a decreasing trend in terms of the detection speed. Different components can be added for practical applications according to the requirement of the actual scenario for fruit detection in different scenarios.

## Conclusion

This paper proposes an efficient and lightweight fruit-detection network model. The algorithm is composed of two main parts. The backbone network adopts Light-CSPNet with a high speed and accuracy; a down-sampling method based on the variation of the feature-map size is employed to compensate for the problem encountered in the conventional single down-sampling method wherein the characteristics of the feature maps at different scales are not considered. The feature-fusion module designs three shallow fusion feature extraction branches for the detection of small-sized fruits. The multiscale fusion of the dual attention module is used to enhance the feature representation and further improve the accuracy of fruit detection. We compare the AP and the detection speed between the conventional single-stage target-detection algorithm YOLO series and the proposed algorithm and tested them on NVIDIA edge devices, namely, Jetson Xavier NX, TX2, and NANO. The detection accuracy can exceed that of the large network models, YOLOv3 and YOLOv4. AP for the orange, tomato, and apple datasets were 0.93, 0.847, and 0.850, respectively, reaching the highest SOTA performance. When the algorithm was deployed on NX, the detection speed for the three datasets reached 21.3, 24.8, and 21.7 FPS, respectively. When the algorithm is deployed on TX2, the detection speed can reach 13.9, 14.1, and 14.5 FPS for the three datasets. When the algorithm is deployed on NANO, the detection speed can reach 6.3, 5.0, and 8.5 FPS, for the three datasets. None of the experiments in this study employed GPU acceleration, model quantization, or any other method, to accelerate the compilation of the target detection model. The proposed algorithm and experimental results could be a guideline for applying target-detection algorithms in the field of horticulture. Different edge equipment can be selected according to the actual requirements, to meet the speed and cost-price requirements. Moreover, the proposed algorithm provides a component add/remove function to afford the flexible adjustment of the model structure, considering the trade-off between the detection accuracy and speed in practical usage. In the future we will try to train and test more fruit datasets in order to better solve the problems of practical applications. We believe that our research can provide a theoretical basis for the development of modern horticulture applications and can be implemented in actual orchards.

## Data Availability Statement

Publicly available datasets were analyzed in this study. This data can be found here: https://www.nature.com/articles/s41438-021-00553-8; https://github.com/laboroai/LaboroTomato#overview.

## Author Contributions

WZ, YL, and WG conceived the ideas and designed the methodology. YL and KC analyzed the data with input of WZ and WG. HL and YD collected the field data. WW and YS supervised the experiment design and manuscript writing. All authors discussed, wrote the manuscript, and gave final approval for publication.

## Conflict of Interest

The authors declare that the research was conducted in the absence of any commercial or financial relationships that could be construed as a potential conflict of interest.

## Publisher’s Note

All claims expressed in this article are solely those of the authors and do not necessarily represent those of their affiliated organizations, or those of the publisher, the editors and the reviewers. Any product that may be evaluated in this article, or claim that may be made by its manufacturer, is not guaranteed or endorsed by the publisher.

## Footnotes

1
https://github.com/I3-Laboratory/orange-dataset

2
https://github.com/laboroai/LaboroTomato#overview
